# Housing Cost Burden and Outcomes Among Medicaid Beneficiaries With Heart Failure

**DOI:** 10.1001/jamahealthforum.2025.5903

**Published:** 2026-01-02

**Authors:** Joniqua N. Ceasar, Lin Yang, Lauren A. Eberly, Ashwin S. Nathan, Eric T. Roberts, Vincent J. Reina, Peter W. Groeneveld, Sameed Ahmed M. Khatana

**Affiliations:** 1 National Clinician Scholars Program, Perelman School of Medicine, University of Pennsylvania, Philadelphia; 2 The Leonard Davis Institute of Health Economics, University of Pennsylvania, Philadelphia; 3 Penn Cardiovascular Outcomes, Quality, & Evaluative Research Center, Perelman School of Medicine, University of Pennsylvania, Philadelphia; 4 Division of Cardiovascular Medicine, Perelman School of Medicine, University of Pennsylvania, Philadelphia; 5 Division of General Internal Medicine, Perelman School of Medicine, University of Pennsylvania, Philadelphia; 6 Center for Healthcare Evaluation, Research, and Promotion, Corporal Michael J. Crescenz Veterans Affairs Medical Center, Philadelphia, Pennsylvania; 7 Department of City and Regional Planning, University of Pennsylvania, Philadelphia

## Abstract

**Importance:**

Housing cost burden is at an all-time high in the US and may disproportionately affect health outcomes among low-income populations. Medicaid-insured individuals and those diagnosed with cardiovascular (CV) disease, such as heart failure (HF), may be especially at increased risk of adverse health outcomes associated with housing cost burden.

**Objective:**

To assess the association between area-level housing cost burden and the probability of CV-related hospitalization or emergency department (ED) visits among Medicaid beneficiaries aged 19 to 64 years with HF.

**Design, Setting, and Participants:**

This cross-sectional study used individual-level health care utilization data obtained from the Transformed Medicaid Statistical Information System Analytic Files (2018-2019). All zip codes in the US with resident Medicaid beneficiaries aged 19 to 64 years who had a preexisting diagnosis of HF and were continuously enrolled in 2019 were included except for those in Alabama, Rhode Island, and Utah due to data quality issues. Data were analyzed from October 2024 to October 2025.

**Exposure:**

Area-level housing cost burden was defined as the zip code–level proportion of housing units occupied by individuals with an annual household income less than $35 000 who spent 30% or more of their income on housing costs.

**Main Outcomes and Measures:**

The probability of a CV-related hospitalization and of a CV-related ED visit in 2019. Generalized estimating equation models were used to evaluate the association between housing cost burden and outcomes after adjusting for individual and area-level factors.

**Results:**

This study included 233 195 individuals (mean [SD] age, 51.5 [9.6] years, 107 447 female [46.1%]) who were living in 19 577 zip codes. The mean (SD) zip code housing cost burden was 67.4% (16.5%). In 2019, 42 886 beneficiaries (18.4%) had at least 1 CV-related hospitalization and 75 392 (32.3%) had an ED visit. After covariate adjustment, a 10–percentage point increase in housing cost burden was associated with higher odds of CV-related hospitalizations (odds ratio [OR], 1.03; 95% CI, 1.01-1.06) and ED visits (OR, 1.03; 95% CI, 1.01-1.04). There were also higher odds of HF-related hospitalizations (OR, 1.04; 95% CI, 1.01-1.07).

**Conclusions and Relevance:**

The findings of this study suggest that area-level housing cost burden may be associated with outcomes among Medicaid beneficiaries with HF and highlights the need to investigate whether strategies that address housing affordability can play a role in improving health outcomes in this population.

## Key Points

**Question:**

Is housing cost burden associated with health care utilization and outcomes among Medicaid beneficiaries with heart failure?

**Findings:**

In this cross-sectional study of 233 195 Medicaid beneficiaries aged 19 to 64 years with heart failure, zip code–level housing cost burden was associated with higher odds of cardiovascular-related hospitalizations and emergency department visits.

**Meaning:**

With the increasing housing cost burden in the US, the findings of this study highlight the need to investigate whether strategies to improve affordability may play a role in improving health outcomes for individuals with lower income and heart failure.

## Teaser

This cross-sectional study assesses the association between housing cost and health care utilization and outcomes among Medicaid beneficiaries aged 19 to 64 years with heart failure.

## Introduction

With housing costs at an all-time high in the US, more than half of renters are cost burdened, spending more than 30% of household income on housing.^1^ Individuals with lower socioeconomic status experience worse health outcomes and are disproportionately impacted by housing costs. Socioeconomic status is associated with cardiovascular (CV) risk and disease, and housing security is an important social determinant of health.^2^ Individuals with CV disease, such as heart failure (HF), may be at risk of adverse health outcomes associated with unaffordable housing, as competing financial needs may interfere with the need for frequent health care encounters, medication adherence, and a healthy diet.^2,3,4^ Additionally, financial strain increases anxiety and stress, which have adverse implications for CV health.^2,5,6,7,8^ These factors may have particularly pronounced implications for Medicaid beneficiaries, a population that predominantly has low income and a high prevalence of CV disease.^9^

The association between housing insecurity and homelessness with poor health outcomes has been well documented; however, how housing cost burden affects health is less well studied.^10,11,12^ Prior studies have demonstrated the association of area-level unaffordable housing with cancer and CV mortality, maternal morbidity, depression, and CV risk factors.^13,14,15,16,17^ Cost-driven residential moves have been associated with greater psychological distress, fewer preventive health visits, and increased emergency department (ED) use.^18,19^ However, most prior analyses have focused on a few municipalities or states, or have not examined individual-level health outcomes. To our knowledge, no previous study has examined the association of area-level housing cost burden with individual-level HF outcomes, especially at more spatially granular levels.

Approximately 6.7 million US adults have HF, with a greater prevalence among those with lower socioeconomic status.^20,21^ HF mortality rates are also increasing, particularly among adults younger than 65 years.^22^ Understanding whether increasing unaffordable housing cost is associated with adverse health outcomes among individuals with HF is essential for both understanding underlying trends, such as the growing income-based disparity in HF prevalence, as well as devising strategies to improve outcomes.^20^ To understand the association of housing cost burden with outcomes among individuals with HF and low income, we evaluated the association of zip code–level housing cost burden and measures of health outcomes and health care utilization among Medicaid beneficiaries aged 19 to 64 years across the US.

## Methods

This study was considered exempt from ethics review under University of Pennsylvania Institutional Review Board guidelines due to secondary research of deidentified routinely collected health care data. Informed consent was waived due to use of deidentified data. This study follows the Strengthening the Reporting of Observational Studies in Epidemiology (STROBE) guideline.^23^

### Data Source and Study Population

Individual health care utilization data were obtained from the 2018 and 2019 Transformed Medicaid Statistical Information System Analytic Files for all Medicaid beneficiaries. The study population was limited to beneficiaries aged 19 to 64 years (as of 2019), with a prior HF diagnosis who were continuously enrolled for all of 2019. Two diagnostic codes for HF on separate occasions were required, with at least 1 in 2018 (eTable 1 in Supplement 1). Beneficiaries with restricted Medicaid coverage (eg, only for pregnancy-related or family planning services) and those who were dual-enrolled in Medicare and Medicaid were excluded due to incomplete utilization data (eMethods 1 in Supplement 1).^24^ Beneficiaries with missing residence information or those with records in multiple states were excluded. All beneficiaries from Alabama and Utah were excluded due to incomplete eligibility data and from Rhode Island because of unavailable residence information. The numbers of people screened and excluded are listed in eFigure 1 in Supplement 1. Based on the Medicaid Data Quality Atlas for inpatient and outpatient claims volume, no states had unusable data.^25,26^ In a sensitivity analysis, states with high concern for inpatient and outpatient claims volume data elements were excluded. In a secondary analysis assessing medication adherence, beneficiaries from states with missing medication copayment data were excluded (Colorado, Idaho, Kentucky, North Carolina, South Carolina, Virginia, and Wyoming).

### Housing Cost Burden

Zip code–level housing cost burden was defined as the proportion of units occupied by residents who have low household income (<$35 000 annually) and pay 30% or more of their income on housing costs. Housing-cost burden data were obtained from the 2019 five-year American Community Survey. In 2019, for a 4-person household, a household income of $35 000 was approximately 136% of the federal poverty level, close to the income eligibility threshold for adult Medicaid beneficiaries aged 19 to 64 years after passage of the Patient Protection and Affordable Care Act (138% of the federal poverty level among Medicaid expansion states).^27^ Data sources for other area-level variables are listed in eMethods 2 in Supplement 1.

### Outcomes and Missing Data

Primary outcomes were the probability of having a CV-related hospitalization and of having a CV-related ED visit in 2019. Other outcomes of interest were the probability of HF-related and all-cause hospitalizations and ED visits. Encounters were identified using diagnostic and procedure codes (eTable 1 in Supplement 1). We also examined the annual number of any-cause and HF-related outpatient visits. Adherence to guideline-recommended medical therapies was assessed for individuals with systolic HF: β-blockers and angiotensin converting-enzyme inhibitors, angiotensin II receptor blockers, or angiotensin receptor–neprilysin inhibitors. Medication adherence was measured using proportion of days covered with a recommended medication during 2019 starting from the first fill date through the end of the year and excluding beneficiaries who were not prescribed these medications.

We used multivariate imputation by chained equations to impute missing beneficiary race and ethnicity data, generating 15 imputed datasets.^28^ Race and ethnicity data included the following categories: Hispanic (all races), non-Hispanic Black, non-Hispanic White, and non-Hispanic other race (Asian, American Indian and Alaska Native, Hawaiian or Pacific Islander, more than 1 race); these categories were combined due to small numbers in each group. These data were obtained from reported values in the Medicaid Transformed Medicaid Statistical Information System and included in the analysis, as prevalence of CV disease differs between different race and ethnicity groups. The imputation model included the same covariates as the primary model and an indicator for CV hospitalization.

### Statistical Analysis

Beneficiary-level demographic and clinical characteristics and zip code–level covariates for the study population were compared across tertiles of zip codes based on housing cost burden. The Pearson correlation coefficient of housing cost burden and other zip code–level covariates was also assessed.

Analysis of variance was used to compare continuous variables, and χ^2^ tests were used to compare categorical variables. Generalized estimating equation (GEE) models were fit to evaluate the association of housing cost burden with the outcomes. The GEE models included the following beneficiary-level covariates: age, sex, race and ethnicity, Medicaid eligibility (disability, Medicaid expansion related, other), and indicators for 29 medical comorbidities based on the Elixhauser Comorbidity Index (based on presence of diagnostic codes in 2019).^29^ Data on race and ethnicity were collected by each individual state Medicaid agency and then submitted to CMS. Administrative data on race and ethnicity are self-reported at the time of application and redetermination. Medicaid eligibility groups were categorized based on Centers for Medicare & Medicaid Services recommendations (eMethods 1 in Supplement 1).^30^ The models accounted for the clustering of beneficiaries within zip codes. State fixed effects accounted for clustering within states. Robust SEs were used. Because housing cost burden is likely to be correlated with other area-level socioeconomic and demographic factors that influence health, we also fit a second set of models including other zip code–level covariates. These models included 17 components of the area deprivation index (ADI).^31^ As the ADI was not available at the zip code level, we used the individual components rather than the composite score. Additional area-level covariates included are listed in eMethods 3 in Supplement 1. While many of these variables are likely to be correlated with each other, in the absence of severe multicollinearity with the primary exposure of interest, including many area-level measures may minimize the likelihood of omitted variable bias.^32^ Multicollinearity was assessed using variance inflation factors and model fit using quasilikelihood information criterion. Additional details of the GEE models are included in eMethods 3 in Supplement 1. Logistic GEE models were fit for binary outcomes, and Poisson models were fit for the number of outpatient visits and the number of days covered with medications as the outcome and log of the number of days in the period as the offset term for adherence. A count-based distribution was used for medication adherence based on prior recommendations.^33^ In a sensitivity analysis, linear models, with proportion of days covered as the outcome, were fit. Medication adherence models also included medication copayment and an indicator of no copayment. GEE models for the primary outcomes were also fit after stratification by zip code metropolitan status.

We conducted a secondary analysis of beneficiaries who moved zip codes (within the same state) between 2018 and 2019. This sample was limited to beneficiaries with continuous Medicaid enrollment in both years and at least 1 HF-related encounter in either year. We leveraged variation in whether individuals moved to zip codes with higher vs lower housing cost burden to estimate the mean within-person effect size of moving to a community with a higher housing cost burden. We controlled for year fixed effects to control for secular trends and beneficiary-level fixed effects to control for time-invariant person-level factors. We included the same zip code–level covariates as the primary analysis and clustered SEs by state. From these models, the estimated effect size of moving to a zip code with a housing cost burden that was 10–percentage points (pps) higher than the person’s original zip code was calculated.

Two-tailed statistical tests were used. *P* < .05 was considered statistically significant. Summary measures are presented as means (SDs) and medians (IQRs), and regression estimates are presented with 95% CI. Analyses were conducted from October 2024 to October 2025 using SAS 9.4 (SAS Institute Inc) and Stata 18 (Stata Corp LLC).

## Results

A total of 233 195 Medicaid beneficiaries aged 19 to 64 years with preexisting HF were identified. These beneficiaries lived in 19 577 zip codes (representing 58.2% of populated zip codes).^34^ The mean (SD) proportion of housing units occupied by residents with low income and high housing cost burden across these zip codes was 67.4% (16.5%). The distribution of housing cost burden across the zip codes is displayed in the Figure and eFigure 2 in Supplement 1. The mean (SD) age of beneficiaries was 51.5 (9.6) years, and the sample included 107 447 females (46.1%) and 125 748 males (53.9%). After dividing the study population based on tertiles of housing cost burden (tertile 1, 0%-59.9%; tertile 2, 60.0%-76.4%; tertile 3, 76.5%-100%) a smaller proportion of beneficiaries were female in the highest tertile vs the lowest tertile (44.0% vs 50.3%; *P* < .001) (Table 1). Compared with the lowest cost burden tertile, the highest cost burden tertile also had a higher proportion of non-Hispanic Black beneficiaries (37.2% vs 15.6%; *P* < .001) and Hispanic beneficiaries (18.3% vs 5.2%; *P* < .001). Summary measures for other individual and zip code–level variables are shown in Table 1. Medical comorbidity prevalences are shown in eTable 2 in Supplement 1. Correlation between zip code–level covariates is shown in eFigure 3 in Supplement 1. The greatest direct correlation of housing cost burden was with median mortgage cost (Pearson correlation coefficient = 0.72; 95% CI, 0.71-0.73) and median gross rent (Pearson correlation coefficient = 0.67; 95% CI, 0.66-0.67). The greatest inverse correlation of housing cost burden was with proportion of residents in rural areas (Pearson correlation coefficient = −0.64; 95% CI, −0.65 to −0.63).

**Figure.**
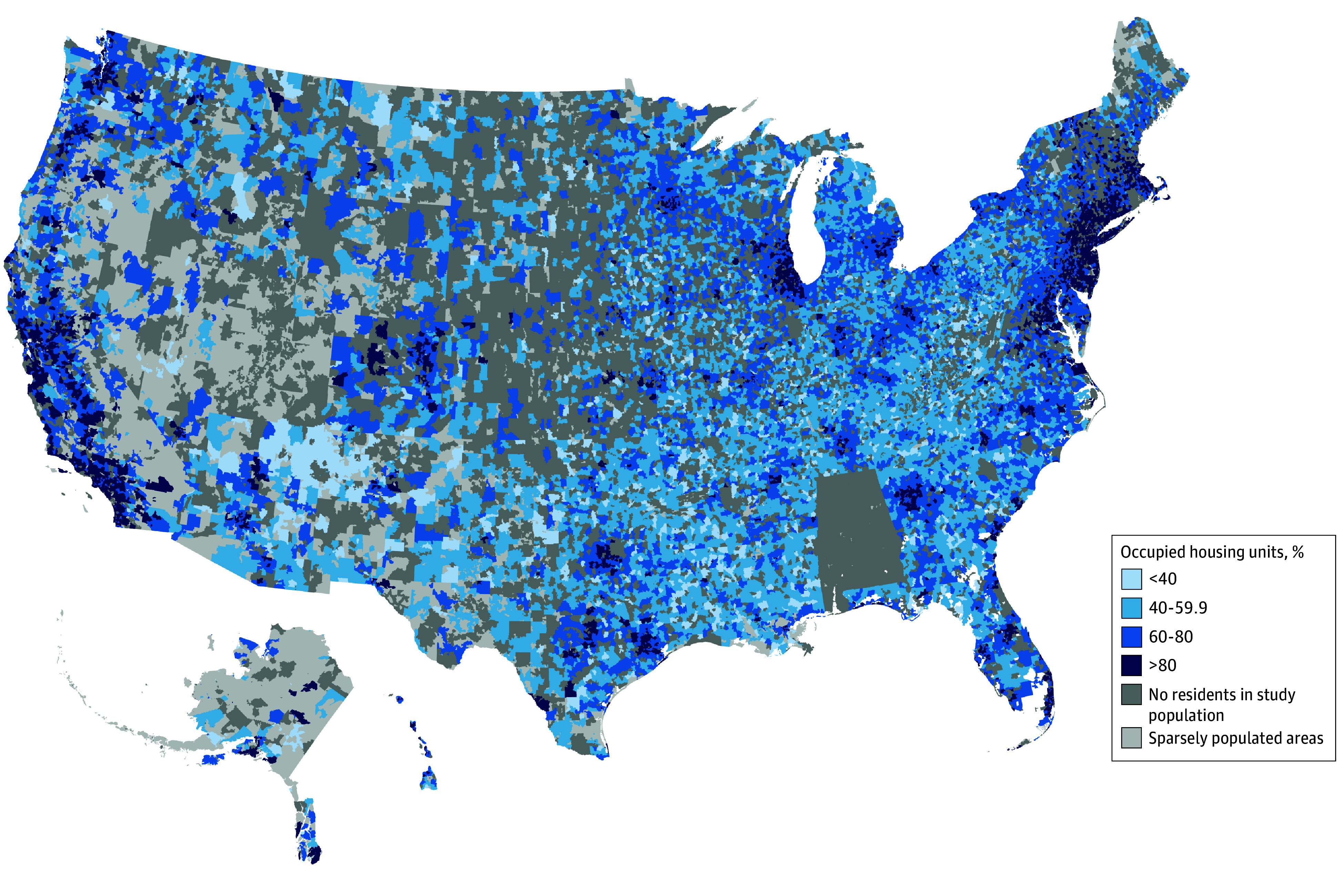
Housing Units Occupied by Individuals With Low-Income and Housing Costs 30% or More of Household Income Stratified by Zip Code Data were obtained from the 2019 five-year American Community Survey. Low income was defined as an annual household income less than $35 000. Zip code tabulation area was used as surrogate for zip code.

**Table 1.** Study Population Characteristics by Tertiles of Zip Code–Level Housing Cost Burden^a^

Characteristic	Population by tertile, mean (SD)			*P* value
	First (n = 27 789 beneficiaries)	Second (n = 87 746 beneficiaries)	Third (n = 117 660 beneficiaries)	
Age, y	51.7 (9.3)	51.5 (9.5)	51.5 (9.7)	.007
Sex, No. (%)				
Female	13 964 (50.3)	41 722 (47.5)	51 761 (44.0)	<.001
Male	13 825 (49.7)	46 024 (52.5)	65 899 (56.0)	
Race and ethnicity, No. (%)				
Hispanic	1144 (5.2)	5843 (7.9)	18 620 (18.3)	<.001
Non-Hispanic Black	3416 (15.6)	25 572 (34.6)	37 766 (37.2)	
Non-Hispanic White	16 066 (73.6)	40 020 (54.2)	37 564 (37.0)	
Non-Hispanic other^b^	1213 (5.6)	2408 (3.3)	7604 (7.5)	
Missing data	1144 (5.2)	5843 (7.9)	18 620 (18.3)	
Medicaid eligibility group, No. (%)^c^				
Persons with disabilities	18 279 (65.8)	54 420 (62.0)	58 573 (49.8)	<.001
Medicaid expansion group	7215 (26.0)	26 167 (29.8)	48 967 (41.6)	
Other	2295 (8.3)	7159 (8.2)	10 120 (8.6)	
Zip code–level variables				
Proportion of housing units occupied by individuals with low income and housing costs ≥30% of income	49.4 (7.7)	68.6 (5.1)	84.2 (5.0)	<.001
Proportion of residents ≥25 y with <9 y of education	5.8 (4.5)	5.5 (4.8)	8.1 (7.0)	<.001
Proportion of residents ≥25 y with at least a high school diploma	83.7 (7.4)	84.8 (8.2)	83.1 (10.6)	<.001
Median household income, $	56 424 (13 788)	60 311 (20 889)	72 175 (29 486)	<.001
Income disparity^d^	1.24 (0.36)	1.25 (0.40)	1.07 (0.39)	<.001
Proportion of households with income <FPL	14.4 (8.3)	16.0 (9.7)	14.0 (8.7)	<.001
Proportion of population with income <150% of FPL	30.8 (11.2)	31.5 (13.3)	27.9 (12.9)	<.001
Proportion of unemployed civilian labor force ≥16 y	6.8 (4.2)	7.5 (4.3)	7.2 (3.5)	<.001
Median home value, $	113 755 (48 736)	158 926 (129 781)	322 525 (214 040)	<.001
Median gross rent, $	681 (148)	857 (221)	1226 (340)	<.001
Median monthly mortgage, $	606 (193)	892 (366)	1467 (503)	<.001
Proportion of owner-occupied housing units	59.6 (11.2)	52.1 (15.6)	46.5 (17.6)	<.001
Proportion of occupied housing units without complete plumbing	0.9 (2.7)	0.5 (1.1)	0.4 (0.6)	<.001
Proportion of single-parent households with children <18 y	6.6 (3.3)	8.9 (4.4)	8.9 (4.4)	<.001
Proportion of households without a motor vehicle	2.7 (2.7)	2.4 (1.6)	2.1 (1.3)	<.001
Proportion of households without a telephone	7.7 (6.4)	12.8 (12.6)	15.0 (16.0)	<.001
Proportion of households with >1 person per room	2.7 (3.3)	3.0 (2.9)	6.5 (6.1)	<.001
Proportion of residents with income <150% of FPL who use public transportation	1.1 (3.5)	7.0 (12.3)	13.9 (17.8)	<.001
Proportion of residents aged 19-64 y with income <137% of FPL and Medicaid insurance	21.2 (10.8)	22.6 (12.1)	22.6 (12.2)	<.001
Proportion of residents living in rural areas^e^	69.8 (34.8)	21.4 (30.7)	4.2 (13.9)	<.001
Primary care practitioners per 100 000 residents^f^	73.1 (21.4)	73.3 (19.1)	77.5 (19.8)	<.001
Hospital beds per 100 000 residents^f^	2.78 (1.13)	2.28 (0.66)	1.94 (0.54)	<.001
Rural-urban commuting area codes, No. (%)^g^				
Metropolitan areas	10 276 (37.0)	67 705 (77.2)	114 500 (97.3)	<.001
Micropolitan areas	7283 (26.2)	13 523 (15.4)	2421 (2.1)	
Small towns	6166 (22.2)	4612 (5.3)	485 (0.4)	
Rural areas or areas not coded	4064 (14.6)	1906 (2.2)	254 (0.2)	

Abbreviation: FPL, federal poverty level.

^a^ Housing cost burden was defined as the proportion of housing units occupied by low-income households (<$35 000 annually) with housing costs 30% or more of income. Data source: the 2019 five-year American Community Survey unless otherwise specified.

^b^ The following categories were combined due to low numbers: American Indian and Alaska Native, Asian, Hawaiian or Pacific Islander, and more than 1 race.

^c^ Data on these categories were based on the 2018 and 2019 Transformed Medicaid Statistical Information System Analytic Files eligibility file. Additional details on each group are provided in eMethods 1 in Supplement 1.

^d^ Measured as the ratio of households with less than $10 000 annual income to households with $50 000 or more annual income.

^e^ Based on the 2020 US Census.

^f^ Based on the Dartmouth Health Atlas Health Service Area cross-walked to zip code.

^g^ Based on 2010 US Department of Agriculture data.

### Primary Outcomes

In 2019, 42 886 beneficiaries (18.4%) had at least 1 CV-related hospitalization and 75 392 (32.3%) had a CV-related ED visit. After determining the model with the best fit, housing cost burden had only moderate levels of multicollinearity with the other covariates (variance inflation factor, 4.7) (eTable 3 in Supplement 1). In the GEE model with only beneficiary-level covariates, a 10-pp increase in housing cost burden was associated with higher odds of CV-related hospitalization (odds ratio [OR], 1.03; 95% CI, 1.01-1.04; *P* < .001) and ED visits (OR, 1.03; 95% CI, 1.02-1.04; *P* < .001) (Table 2). After adjusting for zip code–level covariates, the regression coefficients were similar. In the fully adjusted model, a 10-pp increase in housing cost burden was associated with an increase of 0.03 (95% CI, 0-0.01) pp in the probability of a CV-related hospitalization and an increase of 0.04 (95% CI, 0-0.01) pp in the probability of a CV-related ED visit. An increase in the housing cost burden from the mean of the lowest cost burden tertile to the highest cost burdened tertile of zip codes (49.4% for tercile 1 to 84.2% for tertile 3) was associated with a higher probability of CV-related hospitalizations (absolute difference, 1.1 [95% CI, 0.3-2.0] pp) and ED visits (absolute difference, 1.3 [95% CI, 0.3-2.4] pp).

**Table 2.** Generalized Estimating Equation Analysis of Change in Odds of Outcomes Associated With a 10–Percentage Point Increase in Zip Code–Level Housing Cost Burden^a^

Outcome	Model adjusted for beneficiary-level covariates and state fixed effects^b^		Model adjusted for beneficiary-level and zip code–level covariates and state fixed effects^b^	
	OR (95% CI)	*P* value	OR (95% CI)	*P* value
Primary^c^				
CV-related hospitalizations	1.03 (1.01-1.04)	<.001	1.03 (1.01-1.06)	.01
CV-related ED visits^d^	1.03 (1.02-1.04)	<.001	1.02 (1.01-1.04)	.01
Secondary outcomes^c^				
HF-related hospitalizations	1.05 (1.03-1.07)	<.001	1.04 (1.01-1.07)	.01
HF-related ED visits^d^	1.02 (1.01-1.04)	<.001	1.01 (0.99-1.04)	.25
All-cause hospitalizations	1.03 (1.02-1.04)	<.001	1.02 (1.00-1.04)	.10
All-cause ED visits^d^	1.00 (0.99-1.01)	.63	1.01 (0.99-1.03)	.37

Abbreviations: CV, cardiovascular; ED, emergency department; HF, heart failure; OR, odds ratio.

^a^ Housing cost burden was defined as the proportion of housing units occupied by low-income households (<$35 000 annually) with housing costs 30% or more of income. Based on 2019 five-year American Community Survey data.

^b^ Details of these models are provided in the eMethods 3 in Supplement 1.

^c^ CV disease–related and HF-related hospitalizations and ED visits were identified as encounters with relevant diagnostic codes (eTable 1 in Supplement 1).

^d^ Encounters with *Current Procedural Terminology* codes 99281 to 99285, revenue center codes 0450 to 0459 and 0981, or place of service code 23.

A sensitivity analysis that excluded states with data quality concerns had estimates similar to the primary model (eTable 4 in Supplement 1). In the metropolitan status–stratified models, among beneficiaries in metropolitan areas, estimates were similar to the primary model (eTable 4 in Supplement 1). There was a nonstatistically significant increase in CV-related hospitalizations and ED visits among beneficiaries in nonmetropolitan areas.

### Secondary Outcomes

There was a significant association between zip code–level housing cost burden and increased odds of an HF-related hospitalization (OR, 1.04; 95% CI, 1.01-1.07) (Table 2). The odds of an HF-related ED visit, all-cause hospitalization, and all-cause ED visit were higher but not statistically significant. Higher housing cost burden was associated with more HF-related outpatient visits (percentage chance, 1.81%; 95% CI, 0.66%-2.96%) but not all-cause outpatient visits (percentage change, 0.34%; 95% CI, −0.33% to 1.02%). Among 165 064 beneficiaries diagnosed with systolic HF, 121 164 (73.4%) were prescribed β-blockers and 107 363 (65.5%) were prescribed an angiotensin-converting enzyme inhibitor, angiotensin II receptor blocker, or angiotensin receptor–neprilysin inhibitor; of these beneficiaries, 105 955 (87.5%) and 94 253 (87.8%) had no medication copayment. In the GEE models, there was no association between housing cost burden and medication adherence (Table 3). A linear GEE model had estimates similar to the Poisson GEE adherence model (eTable 4 in Supplement 1).

**Table 3.** Generalized Estimating Equations Model Analysis of Percentage Change in Outcomes Associated With a 10–Percentage Point Increase in Zip Code–Level Housing Cost Burden^a^

Outcome	Model adjusted for beneficiary-level covariates and state fixed effects^b^		Model adjusted for beneficiary-level and zip code–level covariates and state fixed effects^b^	
	Percentage change (95% CI)	*P* value	Percentage change (95% CI)	*P* value
All-cause outpatient visits^c^	0.13 (−0.23 to 0.54)	.56	0.34 (−0.33 to 1.02)	.33
HF outpatient visits^d^^,^^c^	2.21 (1.50 to 2.91)	<.001	1.81 (0.66 to 2.96)	.02
Medication adherence among beneficiaries with systolic HF^e^				
β-Blockers	0.03 (−0.18 to 0.23)	.80	0.07 (−0.25 to 0.39)	.67
ACEIs, ARBs, and ARNIs	−0.04 (−0.26 to 0.19)	.74	0.18 (−0.19 to 0.56)	.34

Abbreviations: ACEIs, angiotensin-converting enzyme inhibitors; ARBs, angiotensin II receptor blockers; ARNIs, angiotensin receptor–neprilysin inhibitors; HF, heart failure.

^a^ Housing cost burden was defined as the proportion of housing units occupied by low-income households (<$35 000 annually) with housing costs 30% or more of income. Based on 2019 five-year American Community Survey data.

^b^ Details of these models are provided in the eMethods 3 in Supplement 1.

^c^ Encounters with *Current Procedural Terminology* codes 99201-99215, 99385-99387, and 99395-99397.

^d^ Cardiovascular and HF hospitalizations and emergency department visits were identified as encounters with relevant diagnostic codes in primary diagnosis (eTable 1 in Supplement 1).

^e^ Systolic HF was identified with relevant diagnostic codes (eTable 1 in Supplement 1). Medication adherence (proportion of days covered) measured starting from first prescription fill date in 2019 to the end of the year.

### Conditional Logistic Regression Model

A total of 20 391 beneficiaries with HF moved between zip codes between 2018 and 2019. In the conditional logistic regression model, a 10-pp increase in housing cost burden was associated with significantly higher odds of CV-related hospitalizations (OR, 1.04; 95% CI, 1.01-1.07) and all-cause hospitalizations for (OR, 1.02; 95% CI, 1.00-1.04) (Table 4). There was no association with higher odds of HF-related hospitalizations (OR, 1.02; 95% CI, 0.98-1.05) or ED visits.

**Table 4.** Conditional Logistic Regression Analysis of Change in Outcomes Associated With a 10–Percentage Point Increase in Zip Code–Level Housing Cost Burden Among Beneficiaries Moving Between Zip Codes of Residence Between 2018 and 2019^a^

Outcome	OR (95% CI)	*P* value
CV-related hospitalizations^b^	1.04 (1.01-1.07)	<.001
CV-related ED visits^b^	1.00 (0.97-1.02)	.76
HF-related hospitalizations	1.02 (0.98-1.05)	.35
HF-related ED visits	1.00 (0.97-1.02)	.87
All-cause hospitalizations	1.02 (1.00-1.04)	.04
All-cause ED visits	1.02 (0.96-1.07)	.55

Abbreviations: CV, cardiovascular; ED, emergency department; HF, heart failure; OR, odds ratio.

^a^ Housing cost burden is defined as the proportion of housing units occupied by low-income households (<$35 000 annually) with housing costs 30% or more of income. Based on 2019 five-year American Community Survey data. Study population limited to beneficiaries who moved residence between 2018 and 2019 (within the same state), were continuously enrolled throughout 2018 and 2019, and had at least 1 heart failure encounter in 2018 and 2019. Conditional logistic regression with the same zip code–level covariates as generalized estimating equations models, year fixed effects, and state-clustered SEs.

^b^ Cardiovascular and HF hospitalizations and ED visits were identified as encounters with relevant diagnostic codes in primary diagnosis (eTable 1 in Supplement 1). ED visits were identified as encounters with *Current Procedural Terminology* codes 99281-99285, revenue center codes 0450-0459, and 0981, or place of service code 23.

## Discussion

In this analysis of Medicaid beneficiaries aged 19 to 64 years with HF across the US, residence in an area with high rates of housing cost burden was associated with higher odds of hospitalizations and ED visits for CV disease and HF after controlling for individual-level and area-level factors. Among a subgroup of beneficiaries who relocated between zip codes, an increase in housing cost burden was associated with increased CV-related and all-cause hospitalizations.

With an increase in the prevalence of HF coinciding with unaffordable rising housing costs in the US, understanding whether high housing cost burden is associated with health care utilization and outcomes in individuals with HF is important.^35^ Previous studies with limited sample sizes have noted an association between measures of unaffordable housing or cost burden on health utilization and outcomes among different populations.^15,16,18,19^ Another study reported an association between unaffordable county-level housing cost and county-level CV mortality rates, but the study was limited by a lack of individual-level data.^14^ However, these previous studies support the findings of our national analysis, which found an association between area-level housing cost burden and health care utilization and disease outcomes.

This analysis does not elucidate mechanisms by which area-level housing cost burden may be associated with HF outcomes. Housing cost burden disproportionately impacts lower-income households and may result in financial trade-offs or increased psychosocial stress, including an increase in anxiety and depressive symptoms, which may adversely affect CV health.^2,5,36,37^ Dietary factors also play an important role in HF outcomes, and costly housing is associated with lower expenditure on food.^4^ Housing cost burden may also be associated with area-level factors, such as high eviction rates and fewer green spaces, that may have implications for health outcomes.^38,39^

The primary exposure, housing cost burden, is an area-level measure, precluding assessment of individual-level housing cost burden and health outcomes. While many area-level socioeconomic measures, as well as composite indexes, such as the ADI, have been associated with HF health outcomes, this analysis suggests that unaffordable housing is associated with HF health care utilization and outcomes independent of a large number of other such measures.^40,41^ A high proportion of individuals with high housing cost burden in an area may therefore be helpful for identifying communities where residents are especially vulnerable to disease exacerbations requiring greater resource investment, independent of other area-level factors.

Unlike prior studies that have suggested unaffordable housing cost may be related to lower medication adherence, our analysis did not find such an association.^36,42^ This may be due to nearly 90% of beneficiaries having no copayment for the medications analyzed, improving accessibility. Housing cost burden was also associated with more outpatient HF visits, although not with overall outpatient visits. While a higher frequency of outpatient visits for HF care could indicate greater engagement, worsening HF symptoms have also been shown to increase outpatient visits.^43^ Therefore, our findings suggest that residing in an area with a high housing cost burden may be associated with greater severity of disease and the need for higher levels of health care utilization.

Whether measures to improve housing affordability can lead to improvements in health outcomes for Medicaid beneficiaries with HF is unclear. In 1 study of Medicaid beneficiaries, including beneficiaries experiencing homelessness or experiencing unaffordable housing, moving into affordable housing was associated with fewer ED visits.^44^ However, a meta-analysis of various strategies promoting stable and affordable housing, such as rental subsidies, found limited evidence of improved health.^45^ Many housing intervention studies have focused on individuals experiencing housing instability or homelessness, which may limit applicability to the broader population experiencing high housing costs. With the association between psychosocial stress and adverse outcomes, HF could be a condition that may be especially sensitive to the stress of housing cost burden and warrants further investigation into whether policies addressing housing affordability can improve health outcomes among individuals with HF and similar chronic diseases.^5,46^

### Limitations

This study has some limitations. Beneficiaries who disenrolled from Medicaid or died during the study period were excluded to ensure complete claims data. Therefore, this analysis cannot examine an association between housing cost burden and mortality or investigate outcomes of those without continuous enrollment. This limitation may underestimate the magnitude of our study findings, as Medicaid discontinuity has been associated with increased CV-related hospitalizations, and measures such as CV-related and HF-related hospitalizations are associated with increased mortality.^47,48^ Zip codes were used as a surrogate for neighborhoods, as this was the most spatially granular residence information available in the data. However, zip codes are heterogeneous in area and population size and some can cross political boundaries of cities, counties, and (rarely) states. Although our analysis accounted for several individual-level and area-level factors, unmeasured confounding remains possible. We were unable to account for receipt of government housing assistance. Additionally, among individuals who moved, we were unable to determine the specific reason for moving or the number of moves over the study period. Furthermore, zip code–level housing cost burden is based on the 5-year American Community Survey, which assumes static costs over this time frame. Due to incomplete utilization data, many beneficiaries dual-enrolled in Medicare and Medicaid were excluded. Finally, we were unable to make causal inferences due to the study’s cross-sectional design.

## Conclusions

In this cross-sectional study of adult Medicaid beneficiaries aged 19 to 64 years with HF, residing in a zip code with a high housing cost burden was associated with increased odds of CV-related hospitalizations and ED visits. With rising costs of housing in the US, this study highlights the need to investigate whether strategies to improve affordability can play a role in improving health outcomes for individuals with lower income and HF.

## Notes

Supplement 1. **eMethods 1.** Medicaid restricted benefits and eligibility groups **eMethods 2.** Data sources **eMethods 3.** Generalized estimating equations model and covariates **eTable 1.** Codes to identify encounters and cardiovascular diagnoses **eTable 2.** Prevalence of medical comorbidities by tertiles of ZIP code-level housing unaffordability **eTable 3.** Quasi-likelihood information criterion (QIC) for different specifications for GEE models **eTable 4.** Sensitivity and stratified analyses - Change in outcomes associated with a 10–percentage point increase in ZIP code-level housing cost burden **eFigure 1.** Study population **eFigure 2.** Distribution of housing unaffordability across ZIP codes **eFigure 3**. Correlation between different ZIP code-level covariates

Supplement 2.
Data Sharing Statement
