# Supplementary material for: Housing Cost Burden and Outcomes Among Medicaid Beneficiaries With Heart Failure
Source: JAMA Health Forum. 2026 Jan 2;7(1):e255903. doi: 10.1001/jamahealthforum.2025.5903 (PMC12761336; doi:10.1001/jamahealthforum.2025.5903)
Supplement: Supplement 1. — eMethods 1. Medicaid restricted benefits and eligibility groups eMethods 2. Data sources eMethods 3. Generalized estimating equations model and covariates eTable 1. Codes to identify encounters and cardiovascular diagnoses eTable 2. Prevalence of medical comorbidities by tertiles of ZIP code-level housing unaffordability eTable 3. Quasi-likelihood information criterion (QIC) for different specifications for GEE models eTable 4. Sensitivity and stratified analyses - Change in outcomes associated with a 10–percentage point increase in ZIP code-level housing cost burden eFigure 1. Study population eFigure 2. Distribution of housing unaffordability across ZIP codes eFigure 3. Correlation between different ZIP code-level covariates [file jamahealthforum-e255903-s001.pdf]

Supplemental Online Content

Ceasar JN, Yang L, Eberly LA, et al. Housing cost burden and outcomes among Medicaid beneficiaries with heart failure. *JAMA Health Forum*. 2025;7(1): e255903. doi: 10.1001/jamahealthforum.2025.5903

eMethods 1. Medicaid restricted benefits and eligibility groups

eMethods 2. Data sources

eMethods 3. Generalized estimating equations model and covarates

eTable 1. Codes to identify encounters and cardiovascular diagnoses

eTable 2. Prevalence of medical comorbidities by tertiles of ZIP code-level housing unaffordability

eTable 3. Quasi-likelihood information criterion (QIC) for different specifications for GEE models

eTable 4. Sensitivity and stratified analyses - Change in outcomes associated with a 10–percentage point increase in ZIP code-level housing cost burden a, b e

eFigure 1. Study population

eFigure 2. Distribution of housing unaffordability across ZIP codes

eFigure 3. Correlation between different ZIP code level covariates

This supplemental material has been provided by the authors to give readers additional information about their work.

© 2025 Ceasar JN et al. *JAMA Health Forum*.

eMethods 1: Medicaid restricted benefits and eligibility groups

Beneficiaries identified as the following, at any time in 2019, were considered to have restricted Medicaid benefits and were excluded:

- Individual is eligible for Medicaid but only entitled to restricted benefits based on alien status (including illegal entrants and legal entrants during the 5-year waiting period).
- Individual is eligible for Medicaid but only entitled to restricted benefits based on Medicaid dual eligibility status
- Individual is eligible for Medicaid but only entitled to restricted benefits for pregnancy-related services.
- Individual is eligible for Medicaid but only entitled to restricted benefits for reasons other than alien, dual eligibility or pregnancy-related status (e.g. restricted benefits based upon substance abuse, medical needy or other criteria).
- Individual is eligible for Medicaid, but only entitled to receive family planning services
- Individual is eligible for Medicaid and entitled to Medicaid benefits under an alternative package of benchmark-equivalent coverage.
- Individual is eligible for Medicaid and entitled to benefits under a "money follows the person" (MFP) rebalancing demonstration, as enacted by the deficit reduction act of 2005, to allow states to develop community based long term care opportunities.
- Individual's benefit restrictions are unknown.
- Individual is eligible for Medicaid and entitled to benefits under the psychiatric residential treatment facilities demonstration grant program (PRTF), as enacted by the deficit reduction act of 2005. PRTF grants assist states to help provide community alternatives to psychiatric resident treatment facilities for children.
- Individual is eligible for Medicaid and entitled to Medicaid benefits using a health opportunity account (HOA).
- Individual is eligible for separate CHIP Dental Coverage (Supplemental dental wraparound benefit to employer-sponsored insurance).
- Individual is only eligible for Medicaid health insurance premium payment assistance (no additional Title XIX or XXI FFS or managed care wraparound services) in Massachusetts, New Jersey, Oklahoma, or Vermont.
- Individual is eligible for Medicaid but only entitled to receive prescription drug benefits (beginning in 2003)
- Individual is eligible for Medicaid and Medicare but only entitled to receive prescription drug benefits and restricted benefits based on Medicaid dual eligibility status
- Individual is eligible for Medicaid and Medicare but only entitled to receive prescription drug benefits.

Individuals with the following eligibility types, at any time in 2019, were grouped into three groups based on CMS recommendations (https://resdac.org/sites/datadocumentation.resdac.org/files/2025-10/TAF-TechGuide-DE-File.pdf):

Disabled

- Individuals Receiving SSI
- Aged, Blind and Disabled Individuals in 209(b) States
- Individuals Receiving Mandatory State Supplements
- Institutionalized Individuals Continuously Eligible Since 1973
- Blind or Disabled Individuals Eligible in 1973
- Individuals Who Lost Eligibility for SSI/SSP Due to an Increase in OASDI Benefits in 1972
- Individuals Who Would be Eligible for SSI/SSP but for OASDI COLA increases since April, 1977
- Disabled Widows and Widowers Ineligible for SSI due to Increase in OASDI
- Disabled Widows and Widowers Ineligible for SSI due to Early Receipt of Social Security
- Disabled Adult Children
- Qualified Medicare Beneficiaries
- Specified Low Income Medicare Beneficiaries
- Qualifying Individuals
- Aged, Blind or Disabled Individuals Eligible for but Not Receiving Cash Assistance
- Individuals Eligible for Cash Assistance except for Institutionalization
- Individuals Receiving Home and Community Based Services under Institutional Rules
- Optional State Supplement Recipients - 1634 States, and SSI Criteria States with 1616 Agreements
- Optional State Supplement Recipients - 209(b) States, and SSI Criteria States without 1616 Agreements
- Institutionalized Individuals Eligible under a Special Income Level
- Individuals participating in a PACE Program under Institutional Rules
- Individuals Receiving Hospice Care
- Poverty Level Aged or Disabled
- Individuals Eligible for Home and Community-Based Services
- Individuals Eligible for Home and Community-Based Services - Special Income Level
- Medically Needy Aged, Blind or Disabled
- Medically Needy Blind or Disabled Individuals Eligible in 1973
- Working Disabled under 1619(b)
- Qualified Disabled and Working Individuals
- Qualified Disabled Children under Age 19
- Work Incentives Eligibility Group
- Ticket to Work Basic Group
- Ticket to Work Medical Improvements Group
- Family Opportunity Act Children with Disabilities
- Individuals with Mental Health Conditions (expansion group)

© 2025 Ceasar JN et al. *JAMA Health Forum*.

ACA Medicaid Expansion eligibility related

- Adult Group - Individuals at or below 133% FPL, 19-64, newly eligible for all states
- Adult Group - Individuals at or below 133% FPL, 19-64, not newly eligible for non 1905z(3) states
- Adult Group - Individuals at or below 133% FPL, 19-64, not newly eligible parent/caretaker-relative(s) in 1905z(3) states
- Adult Group - Individuals at or below 133% FPL, 19-64, not newly eligible nonparent/caretaker-relative(s) in 1905z(3) states

All others

- Parents and Other Caretaker Relatives
- Transitional Medical Assistance
- Extended Medicaid due to Earnings
- Extended Medicaid due to Spousal Support Collections
- Pregnant Women
- Deemed Newborns
- Infants and Children under Age 19
- Children with Title IV-E Adoption Assistance, Foster Care or Guardianship Care
- Former Foster Care Children
- Optional Coverage of Parents and Other Caretaker Relatives
- Reasonable Classifications of Individuals under Age 21
- Children with Non-IV-E Adoption Assistance
- Independent Foster Care Adolescents
- Optional Targeted Low-Income Children
- Individuals Electing COBRA Continuation Coverage
- Individuals above 133% FPL under Age 65
- Certain Individuals Needing Treatment for Breast or Cervical Cancer
- Individuals Eligible for Family Planning Services
- Individuals with Tuberculosis
- Medically Needy Pregnant Women
- Medically Needy Children under Age 18
- Medically Needy Children Aged 18 through 20
- Medically Needy Parents and Other Caretakers
- Targeted Low-Income Children
- Deemed Newborn
- Children Ineligible for Medicaid Due to Loss of Income Disregards
- Coverage from Conception to Birth
- Children with Access to Public Employee Coverage
- Children Eligible for Dental Only Supplemental Coverage
- Targeted Low-Income

© 2025 Ceasar JN et al. *JAMA Health Forum*.

Information on each beneficiary’s restricted benefits and eligibility group is based on the 2019 TAF Demographic and Eligibility file.

© 2025 Ceasar JN et al. *JAMA Health Forum*.

eMethods 2: Data sources

Covariate	Data Source	Data provided
**Individual-level healthcare utilization**	2018 and 2019 Transformed Medicaid Statistical Information System (T-MSIS) Analytic Files (TAF)	Inpatient, outpatient, and medication prescription claims, demographic information, residence, Medicaid program related covariates (e.g., dual eligibility) for all Medicaid beneficiaries.
**Area-level housing cost burden**	2019 5-year American Community Survey	ZIP code-level proportion of units with low-income occupants (household income under $35,000) who pay at least 30% of their household income on housing costs
**ZIP-code level demographic and economic information**	2019 5-year American Community Survey	Education, income, home value, gross rent, monthly mortgage, home ownership, proportion of housing units that are owned, without complete plumbing, single-parent, without a motor vehicle, without a telephone, with more than one person per room, reliant on public transportation, with Medicaid
**Rural population proportion**	2020 US Census	Proportion of residents living in rural areas
**Healthcare access**	Dartmouth Health Atlas (2011 for PCPs and 2012 for hospitals)	Number of primary care providers and number of hospital beds. The Dartmouth Health Atlas provides information regarding both federally and non-federally employed PCPs in the fields of family practice, internal medicine or pediatrics.
**Metropolitan status**	US Department of Agriculture	ZIP code-level rural-urban commuting area (RUCA) codes classify areas into categories such as metropolitan, micropolitan, small town, and rural/uncoded areas.

© 2025 Ceasar JN et al. *JAMA Health Forum*.

eMethods 3: Generalized estimating equations model and covariates

The following generalized estimating equations (GEE) model was fit:

$$logit(Outcome_{ij}) = \beta_0 + Cost\ Burden_j + \chi_i + \varphi_j + State_i$$

for beneficiary i living in ZIP-code j. χ_i and φ_j refer to a set of individual and ZIP code level covariates and $State_i$ are state fixed-effects. A compound symmetry covariance structure was used to account for clustering of beneficiaries in ZIP codes. For number of outpatient encounters and medication adherence, Poisson models were fit. For the medication adherence models, the number of days covered with medications (starting form initial medication fill in 2019) was the outcome and the total number of days in period (time form initial fill to end of 2019) was the offset term.

Individual covariates:

Age
Sex
Race/ethnicity (Hispanic, Non-Hispanic Black, Non-Hispanic White, Non-Hispanic other race)
Medicaid eligibility category (persons with disabilities, Medicaid expansion group, all others)
Indicators for the following comorbidities:
Acquired immune deficiency syndrome
Alcohol abuse
Arthropathies
Blood loss anemia
Chronic pulmonary disease
Coagulopathy
Deficiency anemias
Depression
Diabetes with chronic complications
Diabetes without chronic complications
Drug abuse
Electrolyte abnormalities
Heart failure
Hypertension, complicated
Hypothyroidism
Liver disease
Lymphoma
Metastatic cancer
Neurologic disorders
Obesity
Paralysis

© 2025 Ceasar JN et al. *JAMA Health Forum*.

Peptic ulcer disease
Peripheral vascular disease
Psychoses
Pulmonary circulation disease
Renal failure
Solid tumor
Valvular disease
Weight loss

ZIP code-level covariates:

Proportion of housing units with low-income occupants (household income <$35,000) spending ≥30% of household income on housing costs
Proportion of 25 and older population with less than 9 years of education
Proportion of 25 and older population with at least a high school diploma
Proportion of 16 and older population in employed in white-collar occupations
Median family income
Income disparity (ratio of households with <$10,000 annual income to households with. ≥$50,000 annual income)
Proportion of families with income below federal poverty level
Proportion of population with income below 150% of federal poverty level
Proportion of 16 and older civilian labor force who are unemployed
Median home value
Median gross rent
Median monthly mortgage
Proportion of owner-occupied housing units
Proportion of occupied housing units without complete plumbing
Proportion of single-parent households with children younger than 18
Proportion of households without a motor vehicle
Proportion of households without a telephone
Proportion of households with more than 1 person per room
Proportion of residents living in rural areas (based on 2020 US Census)
Proportion of 19- to 64-year-old residents with income under 137% of the federal poverty level with Medicaid for health insurance
Number of primary care providers per 100,000 residents (based on Dartmouth Atlas Health Service Area)
Number of hospital beds per 100,000 residents (based on Dartmouth Atlas Health Service Area)
Proportion of residents with income under 150% of the federal poverty level who use public transportation

© 2025 Ceasar JN et al. *JAMA Health Forum*.

2010 Rural-Urban Commuting Areas codes – Metropolitan areas, micropolitan areas, small towns, rural areas/not coded

For medication adherence models, monthly medication copayment and indicator for zero copayments were also included.

© 2025 Ceasar JN et al. *JAMA Health Forum*.

eTable 1: Codes to identify encounters and cardiovascular diagnoses

**Encounter types**	**Current Procedural Terminology® (CPT) and other codes**
Emergency Department Visits	CPT ® codes 99281-99285, Revenue Center codes 0450-0459, 0981 or Place of Service code 23
Outpatient visits [a]	CPT® codes 99201–99215, 99385-99387, 99395-99397
**Diagnoses [b]**	**International Classification of Diseases (ICD) 10th revision diagnostic codes**
Heart failure	I09.81, I11.0, I13.0, I13.2, I25.5, I42.0, I42.4 - I42.9, I43.x, I50.x, I51.81, P29.0
Systolic heart failure	I25.5, I42.0, I42.6-I42.9, I50.2x, I50.4x, I50.82, I51.81
Cardiovascular Disease	I00-I99

a. Heart failure related visit identified if heart failure diagnostic code presence in any position.
b. Two diagnostic codes on separate occasions, with at least once in 2018 used to identify presence of pre-existing heart failure diagnosis.

© 2025 Ceasar JN et al. *JAMA Health Forum*.

eTable 2: Prevalence of medical comorbidities by tertiles of ZIP code-level housing unaffordability

Variable	First tertile (N = 27,789 beneficiaries)	Second tertile (N = 87,746 beneficiaries)	Third tertile (N = 117,660 beneficiaries)
	N (%)		
Acquired immune deficiency syndrome	131 (0.5)	1,219 (1.4)	2,580 (2.2)
Alcohol abuse	1,679 (6.0)	7,217 (8.2)	9,920 (8.4)
Deficiency anemias	5,787 (20.8)	20,685 (23.6)	30,243 (25.7)
Arthropathies	1,552 (5.6)	4,670 (5.3)	5,577 (4.7)
Blood loss anemia	605 (2.2)	2,218 (2.5)	2,947 (2.5)
Chronic pulmonary disease	12,388 (44.6)	36,593 (41.7)	40,503 (34.4)
Coagulopathy	1,402 (5.0)	5,199 (5.9)	7,729 (6.6)
Depression	6,362 (22.9)	21,395 (24.4)	25,506 (21.7)
Diabetes with chronic complications	9,990 (35.9)	31,159 (35.5)	42,386 (36.0)
Diabetes without chronic complications	9,228 (33.2)	29,761 (33.9)	39,681 (33.7)
Drug abuse	3,276 (11.8)	13,665 (15.6)	20,001 (17.0)
Hypertension, complicated	21,356 (76.9)	68,811 (78.4)	91,490 (77.8)
Hypothyroidism	3,738 (13.5)	9,629 (11.0)	11,468 (9.7)
Liver disease	3,087 (11.1)	10,565 (12.0)	14,655 (12.5)
Lymphoma	169 (0.6)	644 (0.7)	948 (0.8)
Electrolyte abnormalities	7,536 (27.1)	25,174 (28.7)	33,082 (28.1)
Metastatic cancer	313 (1.1)	1,025 (1.2)	1,389 (1.2)
Neurologic disorders	4,906 (17.7)	15,471 (17.6)	19,149 (16.3)
Obesity	7,898 (28.4)	26,993 (30.8)	35,028 (29.8)
Paralysis	1,300 (4.7)	4,873 (5.6)	6,756 (5.7)
Peripheral vascular disease	4,021 (14.5)	13,108 (14.9)	17,028 (14.5)
Psychoses	3,281 (11.8)	12,272 (14.0)	15,388 (13.1)
Pulmonary circulation disease	1,159 (4.2)	4,615 (5.3)	6,655 (5.7)
Renal failure	5,062 (18.2)	18,184 (20.7)	26,930 (22.9)
Solid tumor	1,315 (4.7)	4,282 (4.9)	5,347 (4.5)
Peptic ulcer disease	506 (1.8)	1,434 (1.6)	2,007 (1.7)
Valvular disease	5,802 (20.9)	18,448 (21.0)	25,179 (21.4)
Weight loss	1,355 (4.9)	4,979 (5.7)	6,775 (5.8)

© 2025 Ceasar JN et al. *JAMA Health Forum*.

eTable 3: Quasi-likelihood information criterion (QIC) for different specifications for GEE models

Specification	QIC
All continuous variables included as linear terms only	156648
All continuous variables included as linear and quadratic terms only	156644
All continuous variables included as restricted cubic splines (with 3 knots)	156659
All continuous variables, except housing unaffordability, included as linear and quadratic terms only. Housing unaffordability included as linear term only	156644
All continuous variables, except housing unaffordability, included as restricted cubic splines. Housing unaffordability included as linear term only	156661

Logistic regression GEE models with the probability of CVD hospitalization as the outcome.

The model with all continuous variables, except housing unaffordability, as linear and quadratic terms and housing unaffordability as only a linear term was chosen based on lowest QIC value.

© 2025 Ceasar JN et al. *JAMA Health Forum*.

eTable 4: Sensitivity and stratified analyses - Change in outcomes associated with a 10 percentage point increase in ZIP code-level housing cost burden [a,b]

Sensitivity Analysis	Outcome	Odds ratio (95% CI)	p-value
**State exclusion based on Medicaid Data Quality Atlas [c]**	Cardiovascular hospitalizations [d,e]	1.03 (1.01, 1.06)	0.02
	Cardiovascular Emergency Department visits [d,f]	1.02 (1.002, 1.04)	0.03
**Metropolitan stratification [g]**	**Metropolitan areas**		
	Cardiovascular hospitalizations [d]	1.03 (1.01, 1.06)	0.01
	Cardiovascular Emergency Department visits [d]	1.02 (1.002, 1.04)	0.02
	**Non-metropolitan areas [g]**		
	Cardiovascular hospitalizations [d]	1.07 (0.97, 1.19)	0.15
	Cardiovascular Emergency Department visits [d]	1.02 (0.94, 1.10)	0.63
		**Change in PDC (95% CI)**	
**Linear GEE Medication adherence model: Outcome Proportion of days covered among beneficiaries with systolic heart failure [h]**	Beta-blockers	0.10 (-0.15, 0.34)	0.44
	Angiotensin-converting-enzyme inhibitors, Angiotensin II Receptor Blockers, or Angiotensin Receptor-Neprilysin Inhibitors	0.17 (-0.10, 0.45)	0.22

a. Housing cost burden defined as the proportion of housing units occupied by low-income households (income <$35,000) with housing costs ≥30% of income. Based on 2019 5-year American Community Survey data.
b. Models adjusted for individual and ZIP code-level covariates. Details of the GEE models in eMethods 3.
c. Sensitivity analysis excluding states listed as "high concern" in the Medicaid Data Quality Atlas for the relevant outcome variable
d. Cardiovascular and heart failure hospitalizations and ED visits identified as encounters with relevant diagnostic codes in primary diagnosis as listed eTable 1
e. Excluding states with TAF inpatient claims volume identified as "high concern": New York, Oklahoma, New Hampshire, Massachusetts, Connecticut. https://www.medicaid.gov/dq-atlas/landing/topics/single/map?topic=g4m45&tafVersionId=23
f. Excluding states with TAF outpatient claims volume identified as "high concern": Minnesota, New Jersey, Massachusetts. https://www.medicaid.gov/dq-atlas/landing/topics/single/map?topic=g4m47&tafVersionId=23
g. Sensitivity analysis with stratification by ZIP code metropolitan status based on 2010 Rural-Urban Commuting Areas codes
h. Linear GEE model proportion of days covered as a linear variable

© 2025 Ceasar JN et al. *JAMA Health Forum*.

eFigure 1: Study population

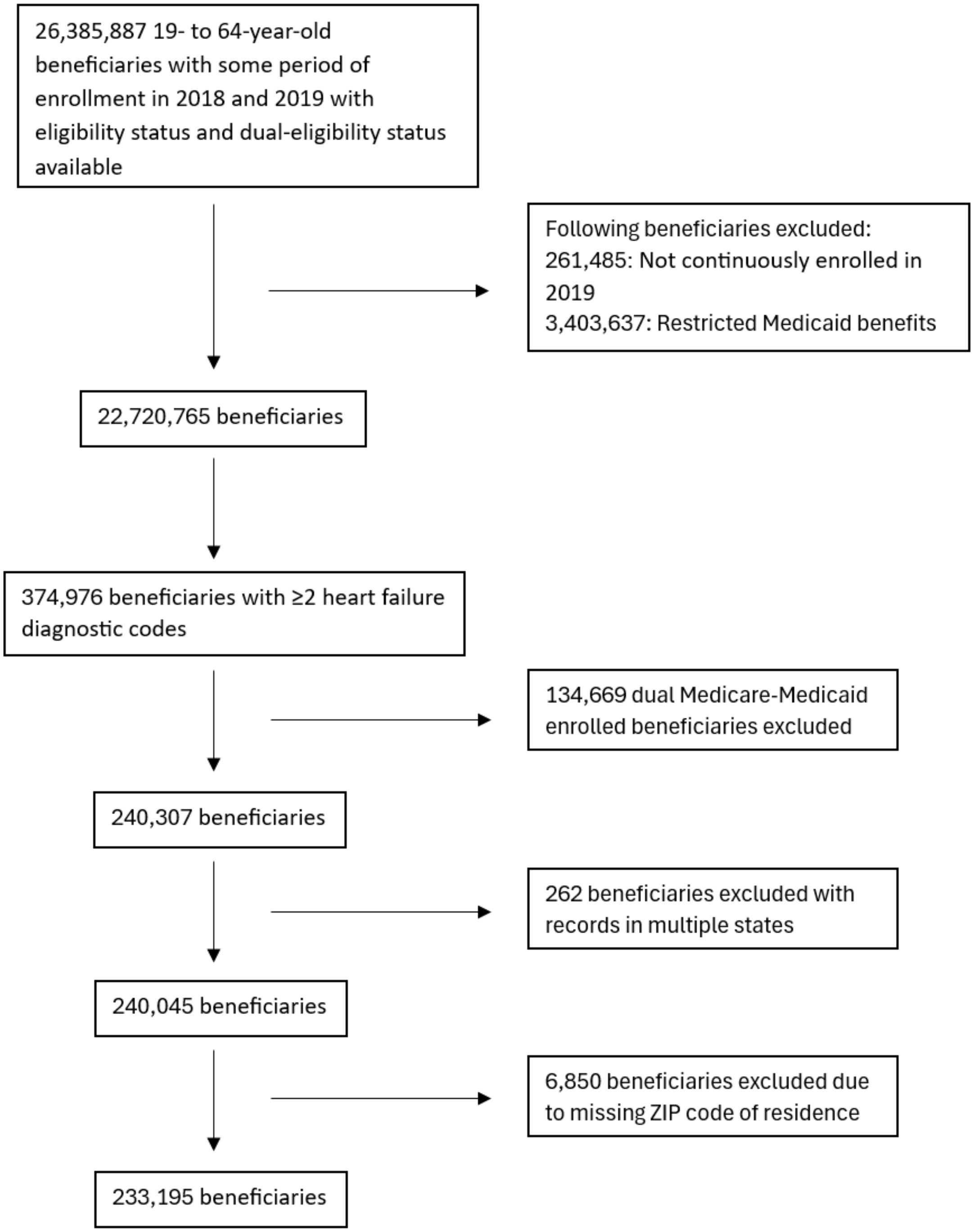

© 2025 Ceasar JN et al. *JAMA Health Forum*.

eFigure 2: Distribution of housing cost burden across ZIP codes

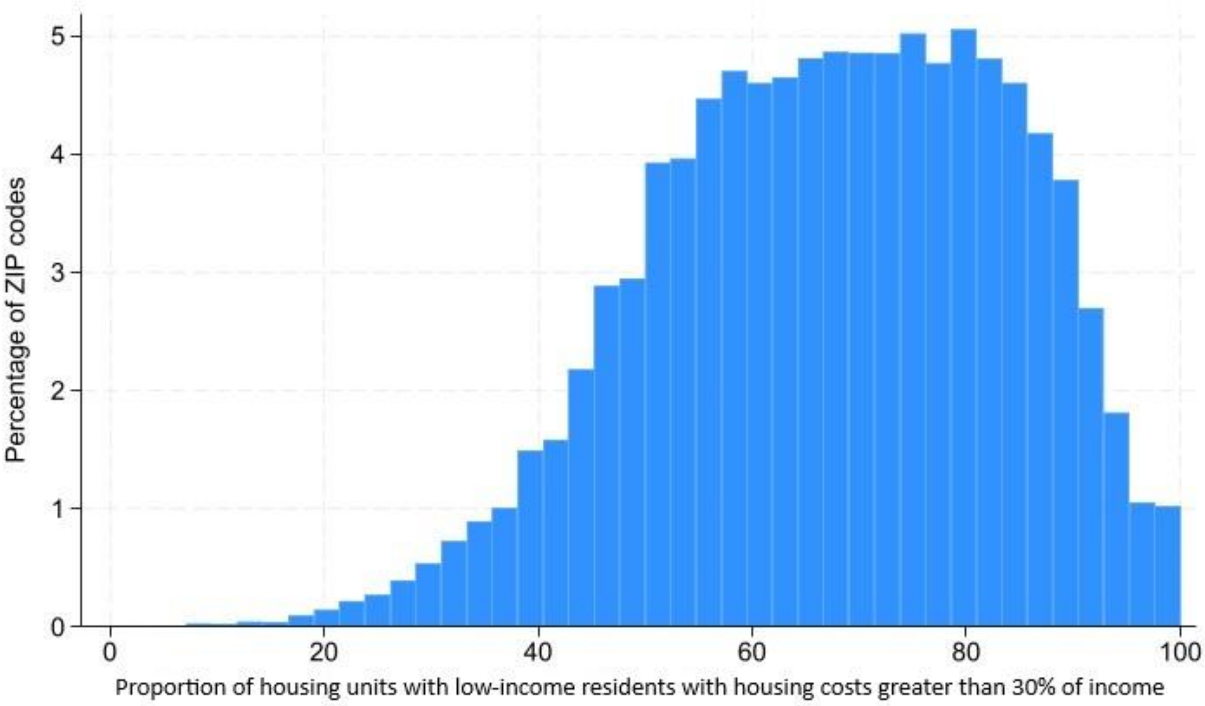

Low-income households defined as household income < 35,000. Based on the 2019 5-year American Community Survey data.

© 2025 Ceasar JN et al. *JAMA Health Forum*.

eFigure 3: Correlation between different ZIP code level covariates

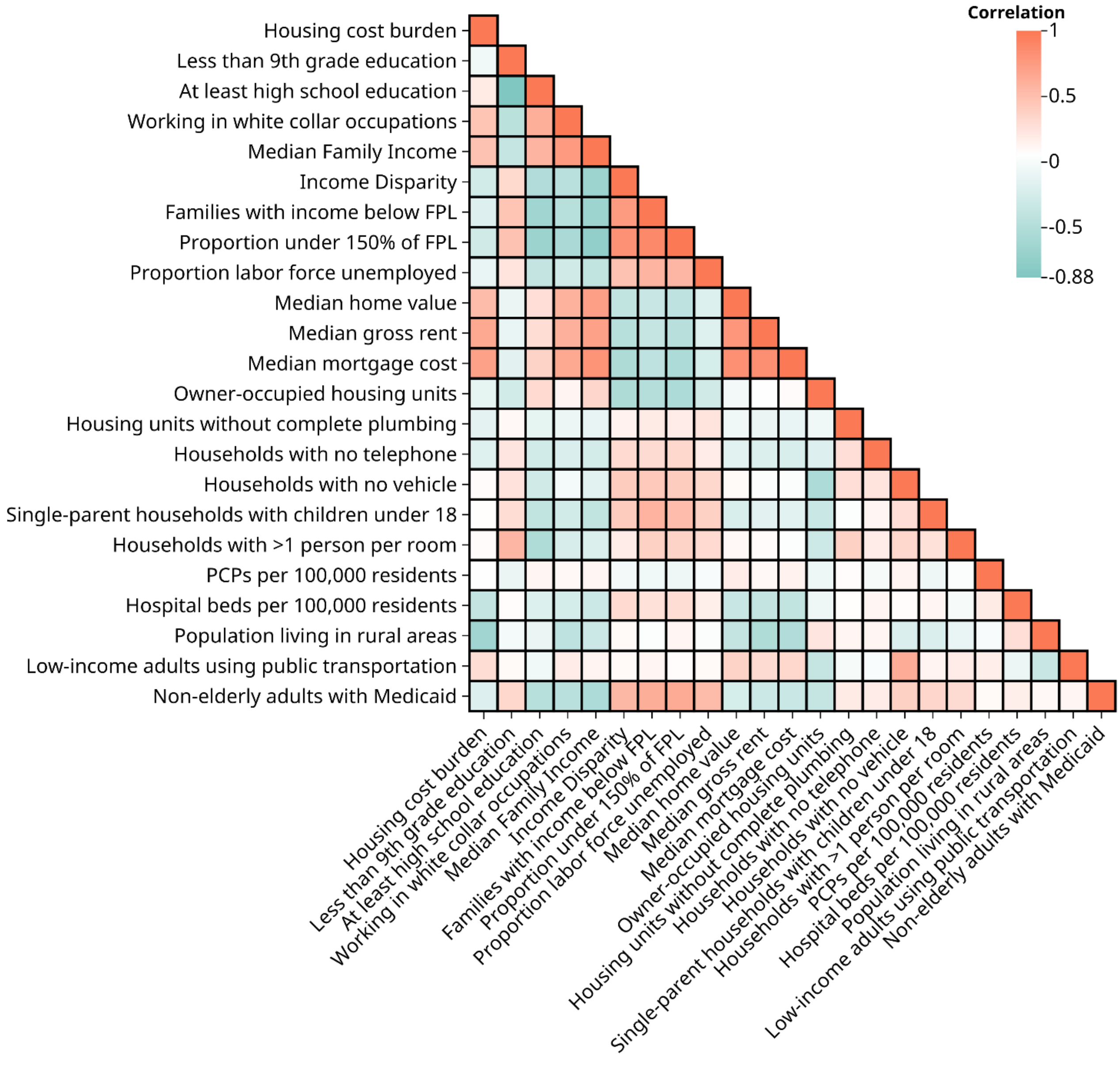

Details of ZIP code level variables in eMethods 3

© 2025 Ceasar JN et al. *JAMA Health Forum*.
