# Supplementary material for: Housing Cost Burden and Outcomes Among Medicaid Beneficiaries With Heart Failure
Source: JAMA Health Forum. 2026 Jan 2;7(1):e255903. doi: 10.1001/jamahealthforum.2025.5903 (PMC12761336; doi:10.1001/jamahealthforum.2025.5903)
Supplement: Supplement 2. — Data Sharing Statement [file jamahealthforum-e255903-s002.pdf]

Data Sharing Statement

Ceasar. Housing Cost Burden and Outcomes Among Medicaid Beneficiaries With Heart Failure. *JAMA Health Forum*. Published January 02, 2026.
doi:10.1001/jamahealthforum.2025.5903

Data
Data available: No
